# Sex-Specific Signature in the Circulating NLRP3 Levels of Saudi Adults with Metabolic Syndrome

**DOI:** 10.3390/jcm10153288

**Published:** 2021-07-26

**Authors:** Nasser M. Al-Daghri, Kaiser Wani, Hind AlHarthi, Amani Alghamdi, Abdullah M. Alnaami, Sobhy M. Yakout

**Affiliations:** 1 Biochemistry Department, College of Science, King Saud University, Riyadh 11451, Saudi Arabia; hindalharthi1@gmail.com (H.A.); aalghamedi@ksu.edu.sa (A.A.); 2 Chair for Biomarkers of Chronic Diseases, College of Science, King Saud University, Riyadh 11451, Saudi Arabia; kwani@ksu.edu.sa (K.W.); aalnaami@ksu.edu.sa (A.M.A.); syakout@ksu.edu.sa (S.M.Y.)

**Keywords:** inflammasome, NLRP3, metabolic syndrome, oxidative stress, inflammatory markers

## Abstract

Recently, inflammasomes such as NLRP3 as cytosolic pattern-recognition receptors have been implicated in the development of inflammation; however, limited investigations report the circulating levels of this protein. The objective, thus, was to investigative circulating NLRP3 levels in Saudi patients with a low-grade inflammatory disorder called metabolic syndrome (MetS). Two hundred Saudi adults aged 30–65, with or without MetS diagnosed on the basis of National Cholesterol Education Programme Adult Treatment Panel III (NCEP ATP III) criteria, were randomly recruited. Five MetS components were established according to the diagnostic criteria in the study subjects. Circulating levels of NLRP3 and known inflammation markers, such as tumor necrosis factor α (TNF-α), C-reactive protein (CRP) and interleukins (IL-1β and IL-18), were measured in the blood samples taken from the study subjects. Gender-based analysis showed a significant elevated circulating levels of NLRP3 in non-MetS men compared to non-MetS females (*p* < 0.001). Moreover, an increase in circulating levels of NLRP3 with a number of MetS components (*p* = 0.038) was observed only in females. A significant positive correlation of NLRP3 levels with age (r = 0.20, *p* = 0.04), BMI (r = 0.32, *p* < 0.01) and waist (r = 0.24, *p* = 0.02) and a significant negative correlation between NLRP3 and HDL-cholesterol (r= −0.21, *p* = 0.03) were also observed in females. Logistic regression analysis also yielded a sex-specific positive association of NLRP3 with MetS in females, with this association influenced mostly by central obesity and dyslipidemia components of MetS. In conclusion, this study suggests a sexual disparity in the circulating levels of NLRP3, with a trend of increasing circulating NLRP3 levels with increasing MetS components observed only in females, influenced mostly by adiposity and dyslipidemia components of MetS. Longitudinal studies with a larger sample size and investigating sex-specific hormones with NLRP3 would be needed to establish a causal relationship of NLRP3 with MetS.

## 1. Introduction

The innate immune response is well-known for its contribution to the inflammatory responses in diseases such as infections, stroke, cardiovascular disease, diabetes and so on [1,2,3]. The identification of germline-encoded pattern recognition receptors (PRR), which recognizes pathogen- and danger-associated molecular patterns (PAMPs and DAMPs), triggers inflammation by stimulating downstream signaling cascades and immune responses initiated in immune cells such as macrophage and dendritic cells [4] PAMPs, such as bacterial endotoxin, are derived from microorganisms, while DAMPs are derived from host cells, including tumor cells, dead cells and compounds produced in response to signals [5]. When these PRRs are present in the cytoplasm, they have been attributed to the detection of endogenous danger signals, which leads to the development of inflammation [6]. One of the best known among these cytoplasmic PRRs is the nucleotide-binding oligomerization domain-like receptor protein 3 (NLRP3), which consists of big multiprotein clusters that get activated by a variety of causes, leading to infection resolution while also having a part in the pathology of cancer [7], inflammatory disorders [8] and autoimmune disorders [9]. Inflammasomes, such as NLRP3, form an arm of the innate immune system by mediating the activation of caspase-1 and pro-inflammatory cytokines (IL-1β and IL-18), leading to a cascade of inflammatory processes which, if unchecked, may result in systemic inflammation, a root-cause of metabolic disorders, such as insulin resistance, diabetes, atherosclerosis, etc. [10,11]. 

Metabolic syndrome (MetS), also known as insulin-resistance syndrome, is a chronic disease of low-grade inflammation that elevates the risk of cardiovascular disease (CVD) and type 2 diabetes mellitus (T2DM) [12]. The prevalence of MetS has escalated globally during the past two decades, especially with Saudi Arabia as one with a high prevalence of 35.7% in adults reported as an average of two diagnostic criteria given by the National Cholesterol Education Programme Adult Treatment Panel III (NCEP ATP III) and International Diabetes Federation (IDF) [13]. MetS patients are more susceptible to develop fatty liver, polycystic ovary syndrome, cholesterol gallstones, hypertension, sleep disorders and cancer, in addition to CVD and T2DM [14]. The individual components of MetS that cluster to form this inflammatory state are insulin resistance (IR), hypertension, central obesity and atherogenic dyslipidemia [15,16]. The pathway linking the pathogenesis of MetS components such as obesity with IR has shown a close association between nutrient excess and immune system activation in most organs related to energy homeostasis since changes in homeostatic parameters induce cells to secrete danger signals involved in inflammation cascade [17,18]. Furthermore, oxidative stress plays a role in the development of MetS, leading to pro-inflammatory and pro-fibrotic pathways [19]. 

Chronic low-grade inflammation, induced by an imbalance in metabolic and immune homeostasis as discussed above, has been implicated as a hallmark for the development of MetS and its associated pathophysiological consequences [16]. Inflammasomes, such as NLRP3, as pattern-recognition receptors, have been implicated in recognizing endogenous danger signals leading to the development of inflammation [20]. There is a growing interest among scientists worldwide in investigating the role of NLRP3 inflammasome activation in the pathogenesis of metabolic disorders, and many reports, including our recent review [21], have helped in understanding NLRP3-mediated adipose tissue inflammation and impairment of insulin signaling pathway leading to IR and MetS. However, most of the literature in this field deals with cytosolic NLRP3 inflammasomes, and limited reports deals with the levels of circulating NLRP3 proteins in humans. Moreover, its association with MetS and its individual components have not been studied. The objective of the current study was to investigate the circulating levels of NLRP3 in Saudi adults with MetS and its association with individual components of MetS. Furthermore, since MetS being a low-grade inflammatory state, it is also interesting to study correlation of circulating levels of NLRP3 with other established circulating pro-inflammatory markers, such as tumor necrosis factor α (TNF-α), C-reactive protein (CRP) and interleukins (IL-1β and IL-18) in the same cohort. 

## 2. Methodology

### 2.1. Subjects and the Study Groups

Two hundred Saudi adults aged 30–65 years were randomly selected from the MetS cohort database of the Chair of Biomarkers of Chronic Disease (CBCD) of the Biochemistry department, College of Science, King Saud University in Riyadh, Saudi Arabia. Subjects who were on anti-hyperglycemic treatment; pregnant or lactating women; and those with known chronic medical conditions, such as renal, hepatic, pulmonary and cardiac diseases, were excluded from the study. A written informed consent form was obtained from all subjects before their inclusion in this study (project # E-20-5369). Ethical approval was obtained from the Ethics Committee of the College of Science Research Center, King Saud University, Riyadh, Saudi Arabia (Ref # 20/0856/IRB). All participants completed a questionnaire on demographic information, general health status and past medical history. Subject with MetS were known cases diagnosed by NCEP ATP III criteria, which classify a person with MetS if three of the following five risk factors or components are present [22].

Central obesity (component 1): waist circumference >101.6 cm in males and >88.9 cm in females.

Hypertension (component 2): systolic blood pressure of >130 mmHg and/or diastolic blood pressure of >85 mmHg or current use of antihypertensive medications.

Hyperglycemia (component 3): fasting glucose level > 5.6 mmol/L.

Low HDL-cholesterol (component 4): HDL-cholesterol <1.03 mmol/L in males and <1.30 mmol/L in females.

Hypertriglyceridemia (component 5): triglyceride level > 1.7 mmol/L.

The subjects were divided into three groups. Group 1 (N = 101) included those with ≤2 MetS components representing the control group (without MetS as per NCEP ATP III criteria); group 2 (N = 49) included the subjects with 3 MetS components and the group 3 (N = 50) included subjects with more than 3 MetS components. Groups 2 and 3 represent the subjects with MetS according to the NCEP ATP III criteria.

### 2.2. Sample Collection and Anthropometrics

Overnight-fasting blood samples were collected by trained technicians and centrifuged to get the serum samples at the recruiting centers; then the samples were transported under suitable temperature to the CBCD laboratory, where they were immediately aliquoted into smaller proportions and stored in freezers until analysis. The anthropometrics included height, weight, and waist and hip circumferences were conducted with routine methods by trained nurses. Mean systolic and diastolic blood pressures (millimeters of Hg) were calculated after being measured twice, using a mercury sphygmomanometer. Body mass index (BMI) and waist–hip ratio (WHR) were calculated by using the following formula: weight in kilograms divided by the square of height in meters for BMI; and quotient between waist and hip circumferences for WHR. 

### 2.3. Biochemical Estimations

Aliquots of serum samples for the recruiting subjects were used to estimate circulating levels of lipid profile; glycemic indices, such as fasting glucose and insulin; 25 (OH) vitamin D; and circulating levels of inflammatory markers such as IL-18, IL-1β, TNF-α, CRP and NLRP3. Glucose, total cholesterol, HDL-cholesterol and triglyceride levels were quantified by using commercially available kits (catalogue nos. 981379, 981812, 981823 and 981301, respectively) in an automated biochemical analyzer (Konelab 20 Thermo-Fischer, Espoo, Finland). The inter-assay CVs for these estimations were ≤5%, ≤3.5%, ≤4% and ≤4.5% for glucose, total cholesterol, HDL-cholesterol and triglyceride assays, respectively. Fasting insulin was measured by Luminex Multiplex (Luminexcorp, Austin, TX, USA), a fluorescent microbead technology, using commercially available kits (catalogue no. HINS-MAG, inter-assay CV ≤ 4.5% between the kits). The glycemic indices HOMA-IR and Quicki were calculated based on the established calculations, using fasting glucose and insulin [23,24,25]. Serum 25(OH) vitamin D was analyzed, using COBAS e-411 autoanalayzer (Roche Diagnostics, Mannheim, Germany) with commercially produced immunoassay kits (IDS Ltd., Boldon Colliery, UK, Reference # 05894913190). 

Circulating levels of IL-1β and TNF-α were quantified by using Flex MAP 3D System (Luminex Corporation, Austin, TX, USA), which utilizes human cytokines Magnetics Bead Panel 1 and 2 (Milliplex Map kit, catalogue nos. HADK1MAG-61K and HADK2MAG-61K). The intra-assay and inter-assay % CV for TNF-α and IL-1β was <10 and <20, and <10 and <15, respectively, according to the manufacturer. Commercially available ELISA assays were used to measure the circulating levels of IL-18 (Quantikine Quickit, QK318, R&D systems, MN, USA) and CRP (K9710s, Immunodiagnostic, AG, Germany). The intra-assay and inter-assay % CV for both of these assays was less than 10% according to the manufacturer. 

Circulating levels of NLRP3 was estimated by commercially available ELISA assay (Cat. #CSB-E15885h, Cusabio, Houston, TX, USA). The minimum detectable dose for this assay was less than 0.039 ng/mL of human NLRP3 according to the manufacturer protocol and the assay had high sensitivity and specificity with CV% of <8% and <10% for intra- and inter-assay precision. The standards and controls used in all the biochemical assays were periodically reviewed by the Quality Assurance team of KSU for reproducible results.

### 2.4. Statistical Analysis

**Figure 1:**
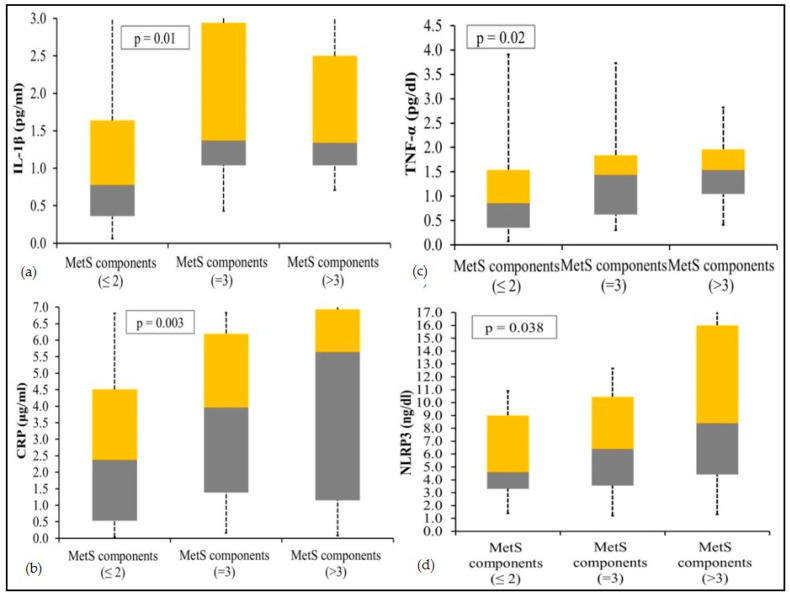
Box-plots showing increasing circulating levels of IL-1β (**a**), CRP (**b**), TNF-α (**c**) and NLRP3 (**d**) with increasing MetS components in females. The yellow and gray boxes show third and first quartile, respectively, and the meeting point signifies the median.

**Figure 2:**
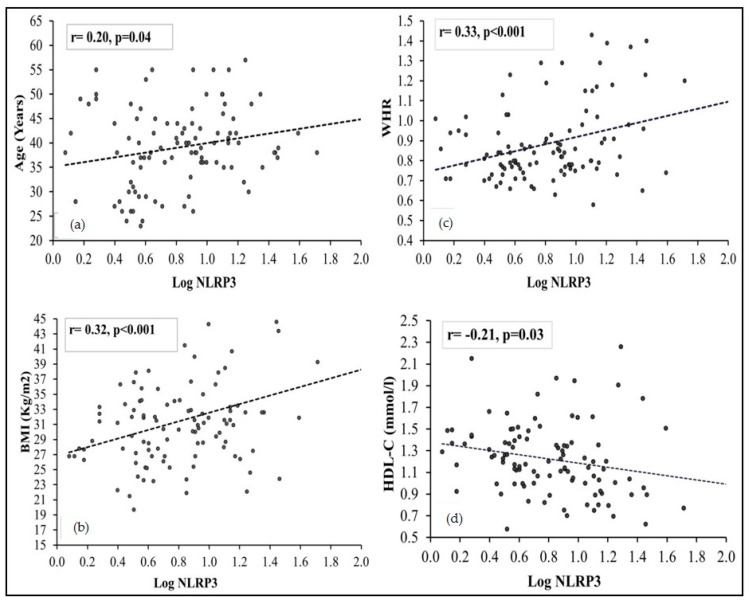
Scatter-plots representing the bi-variate correlation of NLRP3 with age (**a**), BMI (**b**), WHR (**c**) and HDL-cholesterol (**d**) in females. The non-normal continuous variables were normalized by log-transformation before doing the Pearson correlation analysis. The trend-line of the analysis is represented by the black dotted line.

**Figure 3:**
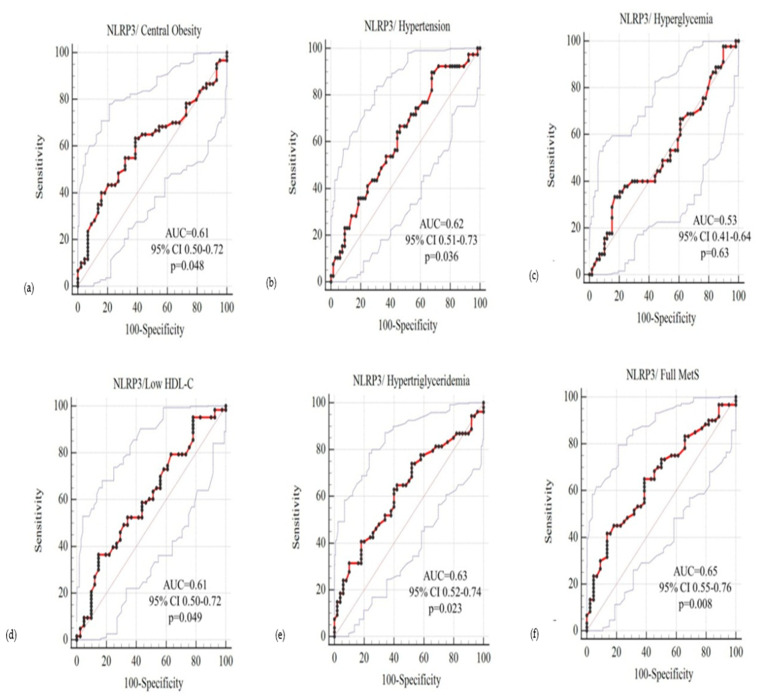
ROC plots for females, using circulating NLRP3 levels as test variable and MetS and its individual components as state variable. AUC is “area under the curve”, 95% CI is 95% confidence interval of AUC, *p* < 0.05 is considered significant. The five components of MetS- central obesity, hypertension, hyperglycemia, low HDL-C, and hypertriglyceridemia; and the full MetS has been plotted in subfigures (**a**–**f**) respectively.

Data were analyzed by using SPSS version 23.0, IBM (SPSS, Chicago, IL, USA). The normal distribution of all the variables in the data was assessed by Kolmogorov–Smirnov test. Central distribution was represented by mean ± standard deviation and median (quartile 1, quartile 3) for continuous normal and non-normal variables respectively; and frequency (%) for categorical variables. To test the differences between the central distributions in the three study groups, ANOVA and Kruskal–Wallis H-test were employed for normal and non-normal variables, respectively. For further analysis, log-transformation was performed to normalize the non-normal continuous variables. The bivariate-associations between circulating NLRP3 levels and other continuous variables were performed by Pearson test and represented by Pearson’s correlation coefficient (r) and associated *p*-value. Furthermore, after looking at the gender-wise differences in circulating NLRP3 levels, the data in the two genders were divided into tertiles based on circulating NLRP3 levels, and a logistic regression analysis was run to assess the odds-ratio of components of MetS (present versus absent) in higher tertiles compared to the lowest tertile. A Univariate model was followed with adjustment with age and BMI in other models. A *p*-value of <0.05 was considered statistically significant. Microsoft excel 2010 was used to plot the figures (Figure 1 and Figure 2), while Figure 3 was plotted by using the help of MedCalc statistical software.

## 3. Results

### 3.1. General Characteristics of the Study Subjects

The study subjects and their baseline characteristics, divided into the three study groups, are summarized in Table 1. The group with MetS components ≤2 is a non-MetS group (N = 101), while the other two groups (Groups 2 and 3) in the table fall into MetS group (N = 99). There was no difference in distribution of genders between the three groups (*p* = 0.53). As expected though, Groups 2 and 3 were significantly older compared to Group 1 (*p* < 0.01). Similarly, BMI, WHR, systolic and diastolic pressures showed an increasing trend with respect to the number of MetS components in the three study groups (all *p* < 0.01). The same trend was followed in glycemic indices (FBG, insulin and HOMA-IR) with a significant constant increase in the number of MetS components in the three study groups, as expected. HDL-cholesterol and the insulin sensitivity index Quicki decreased as the number of MetS components increased in the three groups (*p* < 0.01 for both). The circulating levels of inflammatory markers (TNF-α, CRP and IL-1β) showed an expected trend of elevated levels in groups with higher MetS components (*p* < 0.01 in all); however, for circulating NLRP3 levels, this trend was missing when all subjects were taken into consideration in Table 1 (*p* = 0.44).

### 3.2. Characteristics of the Study Subjects According to Gender

Anthropometric and the biochemical data in the three study groups were further looked at according to the gender, and the results are presented in Table 2. Subjects in groups with higher MetS components were significantly older, and there was no difference in this trend between the genders (*p* < 0.01 in both genders). The anthropometrics, glycemic and lipid indices in both genders almost followed the same trend in the three study groups as seen in the Table 1, and there was no noticeable difference in the trend between the genders. Similarly, the circulating levels of inflammatory markers such as TNF-α, IL-1β and CRP showed an increasing trend with respect to number of MetS components irrespective of the gender. However, when the data were divided between the genders and the circulating levels of NLRP3 checked in the study groups, we found an interesting observation. In males, the circulating levels of NLRP3 seemed to decrease with increase in MetS components but the trend was not statistically significant (*p* = 0.06); however, in females, there was a significant increase in circulating levels of NLRP3 with number of MetS components (*p* = 0.038).

The data were also analyzed between the study groups, MetS (≥3 components) and non-MetS (<3 components); however, this analysis did not change the findings presented in this study. The analysis was presented in the form of Supplementary Materials Tables S1 and S2.

### 3.3. Circulating NLRP3 Levels According to MetS Components

The circulating levels of NLRP3 were checked for individuals with lowest to highest number of MetS components, and the results are presented as Table 3. The circulating levels of NLRP3 followed an increasing trend in individuals with a higher number of MetS components, thus reasserting the sexual disparity seen in the last table. The individuals with all five MetS components were excluded from this analysis, as the group was disproportionate compared to other groups, comprising only 5.5% (*N* = 11) of the total subjects.

The trend of increasing circulating levels of inflammatory markers (TNF-α, CRP and IL-1β) and NLRP3 levels with respect to increasing MetS components in the three study groups in females is depicted in Figure 1. 

### 3.4. Gender-Wise Association of Circulating NLRP3 Levels with Anthropometric and Biochemical Characteristics

The non-normal variables, such as NLRP3, were log-transformed, and a bi-variate correlation analysis was performed, showing an association of NLRP3 with all other variables in different genders; the results are presented in Table 4. In males, there was no statistical significant correlation between NLRP3 and any measured parameter. However, in females, there was a significant positive correlation of NLRP3 levels with age (r = 0.20, *p* = 0.04), BMI (r = 0.32, *p* < 0.01), waist (r = 0.24, *p* = 0.02) and systolic blood pressure (r = 0.22, *p* = 0.02); and a significant negative correlation between NLRP3 and HDL-cholesterol (r= −0.21, *p* = 0.03) was observed in females. 

The bi-variate correlation between NLRP3 and parameters such as age, BMI, WHR and HDL-cholesterol in females is presented as scatter graphs in Figure 2.

### 3.5. Gender-Wise Association of Circulating NLRP3 Levels with Different Components of MetS

Circulating NLRP3 levels in both genders were divided into tertiles, with tertile one and tertile three having the lowest and highest NLRP3 levels, respectively, and a logistic regression analysis was run checking the odds of having different components of MetS in higher tertiles of NLRP3 compared to the lowest tertile. The results of the logistic regression analysis are presented in Table 5. The models used were univariate (Model a), age adjusted (Model b) and age + BMI adjusted (Model c). A gender-specific association of NLRP3 levels with full MetS and its individual components, especially “central obesity” and “low HDL-cholesterol”, was observed only in females in this logistic regression analysis. The logistic regression analysis showed that, in females, higher tertiles of NLRP3 levels were associated with higher odds of getting “central obesity”, as well as “low HDL-cholesterol” component of MetS (p-trend 0.02 and 0.04, respectively), and this statistically significant trend persisted even after adjustment with age and BMI. The other components of MetS except hyperglycemia also showed this trend in females, at least for the univariate model, which suggested increasing hypertriglyceridemia and hypertension with higher tertiles of NLRP3 levels. As for full MetS in females, the univariate analysis suggested that higher tertiles of NLRP3 levels are associated with higher odds of having full MetS (*p*-value for trend = 0.01); however, the statistical significance of the analysis was lost after adjustment with age and BMI. In males, no such significant association of NLRP3 levels with components of MetS or with full MetS was observed.

Receiver operating curves (ROCs) were prepared by using circulating NLRP3 as a test variable to predict the full MetS and its individual five components in females, and the plots are presented in Figure 3.

## 4. Discussion

In this study, we evaluated the circulating levels of NLRP3 in Saudi adults with MetS diagnosed on NCEP ATP III criteria. To the best of the investigators’ knowledge, this was the first report, at least in this population, in terms of determining the association between serum NLRP3 levels and MetS. One of the major findings observed in this study was the sexual disparity in the relationship of MetS with circulating levels of NLRP3. The univariate logistic regression analysis suggested that the odds of having full MetS increased significantly with increasing circulating NLRP3 levels only in case of females. This gender-dimorphic relationship in females was influenced mostly by central adiposity and low HDL-cholesterol components of MetS, possibly confirming the gender-dimorphism theory in the immune response. Moreover, elevated circulating NLRP3 levels, along-with increased IL-1β, CRP and TNF-α levels in subjects with higher MetS components, at least in females, suggests its pro-inflammatory activity.

MetS, a multifactorial pathophysiological disorder with widespread health consequences, is not only characterized by metabolic imbalance but also by an immunologic process in the infiltration of macrophages, T and B cells, etc., in tissues such as adipose, liver and pancreatic islets, resulting in a low-grade inflammation state [26]. When activated, the NLRP3 inflammasome, a cytosolic multiprotein complex, causes caspase-1 to cleave pro-IL-1β and pro-IL-18, resulting in their active forms of pro-inflammatory cytokines involved in the inflammation cascade [27,28,29]. NLRP3 inflammasomes have thus been considered as a link between immune and metabolic processes related to disorders in glucose hemostasis, lipid metabolism and blood pressure [6,30]. NLRP3 inflammasome activation is regulated at both the transcriptional and post-translational level through a two-step classical model of priming and activation, triggered by various DAMPs as a result of metabolic dysfunction [31]. Saturated fatty acids, pro-inflammatory adipokines, excess ATP, reactive oxygen species (ROS), hyperglycemia and other metabolic insults serve as major inducers of a cycle of NLRP3 inflammasome activation and cytokine production [32].

Our data suggest that circulating levels of NLRP3 are positively associated, at least in females, with components of MetS especially central obesity and low-HDL component. Earlier reports show that obese people have increased NLRP3 and IL-1β expression in visceral and subcutaneous deposits, and this has also been confirmed by genetic studies [33,34]. Furthermore, calorie restriction, exercise and weight loss through bariatric surgery have been associated with lower gene expression of NLRP3 and IL-1β, thus suggesting that obesity-induced MetS and NLRP3 inflammasome activity are interrelated [35,36]. Moreover, a significant negative correlation of circulating levels of NLRP3 protein with HDL-cholesterol confirms an anti-inflammatory effect of HDL, which has been attributed to its role in reducing the loss of lysosomal membrane integrity upon phagocytosis of cholesterol crystals [37]. The results from the mentioned studies support the results of our study that suggest that higher circulating NLRP3 levels are associated with MetS components. Some findings, however, report that obesity-mediated inflammation and the production of proinflammatory cytokines in adipose tissue and NLRP3 inflammasome activation are not interdependent [38]; hence, the findings should further be elucidated in future such studies. 

The relationship of NLRP3 inflammasomes with metabolism is gaining increasing attention from last few years, and a number of reports have been published [39,40]. Most of these studies, however, deal with the cytosolic inflammasomes and their role in inflammation. Our data here are important, as limited reports on the circulating levels of NLRP3 are currently in the literature, and our data are probably the first that investigate the association between circulating levels of NLRP3 levels with MetS. Yongfeng Zhang et al., in their study showed that NLRP3 expression in the synovial fluid was positively correlated with arthritis, suggesting its role in the pathogenesis of inflammatory disorders, such as rheumatoid arthritis [41]. Nadine Kerr and colleagues in their recent study demonstrated the potential use of serum inflammasome proteins as biomarkers of stroke [42]. Similarly, a study on the serum levels of NLRP3, published in 2019 by Kuanxue Sun and Hongwei Xia, showed elevated expression in severe blunt abdominal trauma patients and was also correlated with 6-month mortality in these patients [43]. Two more recent studies showed elevated serum NLRP3 levels associated with severity of diseases such as ulcerative colitis [44] and polyradiculoneuropathy [45]. The data from all of these studies are in line with our study, suggesting a potential role of circulating NLRP3 proteins in inflammatory disorders such as MetS. 

The sex-specific differences in circulating levels of NLRP3 observed in this study, with higher levels in males compared to females (median levels of 10.4 and 4.6 ng/dl, respectively, *p* < 0.001 in non-Mets subjects) support the hypothesis that sex differences influence immune responses [46] and that women experience lower rates of chronic inflammatory diseases [47]. An earlier study [48] showed higher mRNA levels of NLRP3 in the peripheral blood mononuclear cells (PBMC) of males compared to females (OR 2.04, 95% CI 1.24–3.35, *p* = 0.03), which corresponds to the higher circulating NLRP3 levels in men observed in this study. Moreover, a sexual disparity was also observed in circulating levels of NLRP3 in subjects with MetS, with significantly higher levels observed with higher MetS components only in females. It would be interesting to study the mRNA levels of NLRP3 in PBMC’s of MetS subjects and investigate whether it follows the same trend as the circulating NLRP3 levels found in this study. This sex-specific differential expression in circulating NLRP3 levels may be attributed in part to the effect of sex hormones, which impact the repertoire of immune response differently in men and women. Studies have shown that decline in estrogen level, particularly after menopause, leads to elevated NLRP3 activation and, hence, higher risk of inflammatory disorders [49]. Most of the female subjects in this study (79.2%) were pre-menopausal, which may explain the low NLRP3 levels compared to men. Further analysis of circulating NLRP3 levels between pre- and post-menopausal women in this study was not possible due to low sample size in post-menopausal group; hence, future large studies on this subject would reveal this distinction in a better manner. Progesterone and androgens such as testosterone, on the other hand, have been linked with NLRP3 inflammasome activation [50]; however, this role of the male sex hormone needs further elucidation, as there are reports which conflict with the assisting role of testosterone for NLRP3 inflammasome activation [51]. Nonetheless, the sex-specific signature observed here in the circulating levels of NLRP3 proteins suggests its active role in the pathogenesis of MetS. 

In this study, a novel sex-specific association between circulating levels of NLRP3 and the status of MetS in Saudi adults was presented. Even though the findings in this study may help in the understanding of the sexual dimorphism in inflammatory response to metabolic disorders, some limitations need to be pointed out. Firstly, the present study, due to nature of its design, was unable to establish a causal relationship of NLRP3 with MetS. Secondly, although the low sample size was enough to give an overview of the association of circulating levels of NLRP3 with MetS, a larger sample size would have provided better results, especially in the elucidation of these findings between pre-menopausal and post-menopausal women. Thirdly, the results may be valid in a specific population, as the homogeneity of the samples used might have influenced the relationship. Additional related studies involving other populations might provide different results, which would be useful, especially considering the scarcity of the literature on the circulating levels of this protein. Lastly, the circulating levels of NLRP3 protein were investigated here without looking at the levels of NLRP3 in PBMC’s, as it may suggest differential regulation of inflammasome activity in PBMC compared to serum.

## 5. Conclusions

In conclusion, this study suggests a sexual disparity in the circulating levels of NLRP3 in Saudi men and women, with a trend of increasing circulating NLRP3 levels with increasing MetS components observed only in females. Moreover, significantly higher NLRP3 levels were observed in non-MetS males compared to females, supporting the gender-dimorphism hypothesis in immune responses. The logistic regression analysis revealed that this differential effect was influenced mostly by adiposity and dyslipidemia components of MetS. Longitudinal studies with a larger sample size would be needed to establish a causal relationship of NLRP3 with MetS. Furthermore, there is a need for future investigations on the association of estrogens and testosterone with the NLRP3 inflammasome and cytokine signaling in MetS, as it may relate to the biologic mechanisms underlying the sexual dimorphism found in this study.

## Figures and Tables

**Table 1:** Anthropometric and biochemical characteristics of the study subjects.

Parameters	Group 1 MetS Components (≤2)	Group 2 MetS Components (=3)	Group 3 MetS Components (>3)	*p*
N (F/M)	101 (50/51)	49 (29/20)	50 (27/23)	0.53
Age (years)	35.49 ± 8	41.57 ± 6.7	42.3 ± 7.9	**<0.01**
**Anthropometrics**				
BMI (kg/m^2^)	28.3 ± 6.6	32.12 ± 5.1	31.64 ± 5.2	**<0.01**
Waist (cm)	87.43 ± 14.7	103.04 ± 17.5	108.3 ± 12.9	**<0.01**
WHR	0.84 ± 0.1	0.94 ± 0.1	1.04 ± 0.2	**<0.01**
Systolic (mmHg)	116.63 ± 12	125.2 ± 14.8	135.88 ± 17.7	**<0.01**
Diastolic (mmHg)	71.01 ± 9.4	75.55 ± 10.3	82.36 ± 11.2	**<0.01**
**Circulating biochemical profile**				
Cholesterol (mmol/L)	5.22 ± 0.9	5.41 ± 1.5	5.51 ± 1.7	0.40
FBG (mmol/L)	5.23 ± 0.9	6.58 ± 3	7.79 ± 3.5	**<0.01**
HDL-Cholesterol (mmol/L)	1.24 ± 0.3	1.12 ± 0.3	0.92 ± 0.2	**<0.01**
Triglyceride (mmol/L)	1.19 (0.9, 1.5)	2.22 (1.5, 2.6)	2.4 (2, 3.4)	**<0.01**
Vitamin D (nmol/L)	42.39 (28.5, 59.8)	47.21 (31.1, 71.9)	35.51 (24.3, 58.9)	0.23
Insulin (µU/mL)	8.06 (4.2, 14.4)	15.26 (4.8, 36.8)	19.05 (10, 45.6)	**<0.01**
HOMA-IR	1.75 (1, 3.8)	4.61 (1.5, 9.6)	8.38 (2.8, 11.8)	**<0.01**
Quicki	0.63 (0.5, 0.7)	0.50 (0.4, 0.7)	0.44 (0.4, 0.6)	**<0.01**
IL-18(pg/mL)	64.09 (36.6, 88.6)	49.29 (33.3, 82.3)	48.25 (37.7, 71.6)	0.33
TNF-α (pg/mL)	0.46 (0.2, 1.2)	1.46 (0.8, 1.8)	1.36 (0.9, 1.8)	**<0.01**
IL-1β (pg/mL)	0.5 (0.4,0.9)	1.37 (1,2.9)	1.31 (1, 2.5)	**<0.01**
CRP (µg/mL)	2.01 (0.59,4.2)	3.96 (1.5,6.2)	5.28 (1.9, 6.2)	**<0.01**
NLRP3 (ng/dl)	8.65 (3.9,11.2)	5.8 (3.8,11.1)	6.5 (4.4, 12.8)	0.44

Note: Data are presented as frequency, mean ± standard deviation and median (Q1, Q3) for categorical, normal continuous and non-normal continuous variables, respectively. The statistical differences in each variable between the three groups, calculated by appropriate statistical tests, are presented as *p*-value. Statistically significant *p*-values (<0.05) has been indicated by bold font. BMI, WHR, FBG, HDL, HOMA-IR represent body mass index, weight by height ratio, fasting blood glucose, high-density lipoprotein and Homeostatic Model Assessment of Insulin Resistance respectively.

**Table 2:** Anthropometric and biochemical characteristics of the study groups on gender basis.

Parameters	MetS Components (≤2) (*N* = 51)	MetS Components (=3) (*N* = 20)	MetS Components (>3) (*N* = 23)	*p*	MetS Components (≤2) (*N* = 50)	MetS Components (=3) (*N* = 29)	MetS Components (>3) (*N* = 27)	*p*
	Males (94)				Females (106)			
Age (years)	35.27 ± 7.8	41.45 ± 5.8	41.87 ± 8.3	**<0.01**	35.7 ± 8.2	41.66 ± 7.3	42.67 ± 7.6	**<0.01**
BMI (kg/m^2^)	26.61 ± 5.6	31.74 ± 5.3	29.86 ± 3.2	**<0.01**	30.03 ± 7	32.38 ± 5	33.14 ± 6.2	0.08
Waist (cm)	92.04 ± 16.9	109.19 ± 16.1	111.43 ± 14.3	**<0.01**	82.72 ± 10.2	98.81 ± 17.4	105.87 ± 11.4	**<0.01**
WHR	0.91 ± 0.1	0.98 ± 0.1	1.02 ± 0.1	**<0.01**	0.76 ± 0.1	0.92 ± 0.2	1.05 ± 0.2	**<0.01**
Sys (mmHg)	119.8 ± 12.3	126.5 ± 11.1	133.96 ± 11.5	**<0.01**	113.4 ± 10.9	124.31 ± 17	137.52 ± 21.7	**<0.01**
Dias (mmHg)	69.69 ± 9.4	74 ± 10.6	78.83 ± 9.6	**0.001**	72.36 ± 9.4	76.62 ± 10.1	85.37 ± 11.7	**<0.01**
Cholesterol (mmol/L)	5.24 ± 0.9	5.37 ± 1.4	5.46 ± 1.7	0.77	5.2 ± 1	5.44 ± 1.7	5.56 ± 1.7	0.52
FBG (mmol/L)	5.22 ± 0.5	6.52 ± 2.2	8.84 ± 4.3	**<0.01**	5.23 ± 1.2	6.62 ± 3.5	6.9 ± 2.4	**<0.01**
HDL-C (mmol/L)	1.13 ± 0.2	0.96 ± 0.2	0.8 ± 0.2	**<0.01**	1.34 ± 0.3	1.23 ± 0.4	1.01 ± 0.3	**<0.01**
Triglyceride (mmol/L)	1.33 (0.9, 1.7)	2.46 (1.8, 2.9)	2.42 (2, 3.5)	**<0.01**	1.07 (0.8, 1.5)	1.97 (1.4, 2.5)	2.38 (2, 2.8)	**<0.01**
Vitamin D (nmol/L)	44.97 (31.6, 61.5)	40.71 (27.1, 56.7)	36.01 (23.5, 55.3)	0.12	33.28 (23, 56.2)	55.07 (35.5, 89.1)	35.51 (24.3, 67.3)	**0.03**
Insulin (µU/mL)	11.2 (6.1, 17.7)	13.23 (4.8, 52.6)	24.3 (7.1, 51.5)	0.19	5.72 (3.4, 10.8)	17.28 (6.8, 32.3)	18.11 (10.2, 42.5)	**<0.01**
HOMA-IR	2.77 (1.6, 4.2)	3.48 (1.5, 11.3)	9.04 (2, 16.3)	**0.02**	1.33 (0.7, 2.4)	4.62 (1.5, 7.9)	6.22 (2.8, 11.6)	**<0.01**
Quicki	0.56 (0.5, 0.6)	0.53 (0.4, 0.7)	0.43 (0.4, 0.6)	**0.02**	0.68 (0.6, 0.8)	0.5 (0.4, 0.7)	0.47 (0.4, 0.6)	**<0.01**
IL-18 (pg/mL)	40.07 (26, 63.5)	54.05 (37, 91.3)	56.84 (40.3, 72.5)	**0.03**	77.59 (61.9, 100.8)	40.79 (31.4, 66.9)	42.95 (34.5, 65.6)	**<0.01**
TNF-α (pg/mL)	0.21 (0.1, 0.3)	1.50 (0.8, 1.6)	1.14 (0.9, 1.7)	**<0.01**	0.86 (0.4, 1.5)	1.44 (0.6, 1.8)	1.54 (1, 1.9)	**0.02**
IL-1β (pg/mL)	0.43 (0.2, 0.6)	1.53 (1.2, 2.4)	1.31 (1.1, 4.7)	**<0.01**	0.78 (0.4, 1.6)	1.37 (1, 2.9)	1.34 (1, 2)	**0.01**
CRP (µg/mL)	1.81 (0.74, 4.09)	4.13 (1.8, 6.4)	5.1 (2.0, 6.1)	**<0.01**	2.37 (0.5, 4.5)	3.96 (1.4, 6.2)	5.64 (1.1, 6.9)	**0.003**
NLRP3 (ng/dl)	10.4 (8.3, 11.4)	5.4 (4, 12)	5.7 (4.4, 11.3)	0.06	4.6 (3.3, 9)	6.4 (3.6, 10.5)	8.4 (4.4, 16)	**0.038**

Note: Data are presented as frequency, mean ± standard deviation and median (Q1, Q3) for categorical, normal continuous and non-normal continuous variables, respectively. The statistical differences in each variable between the three groups, calculated by appropriate statistical tests, are presented as *p*-value. Statistically significant *p*-values (<0.05) has been indicated by bold font. BMI, WHR, FBG, HDL, HOMA-IR represent body mass index, weight by height ratio, fasting blood glucose, high-density lipoprotein and Homeostatic Model Assessment of Insulin Resistance respectively.

**Table 3:** NLRP3 levels according to MetS components.

MetS Components	0	1	2	3	4
All subjects (*N* = 200)					
NLRP3 (ng/dL)	7.9 (3.4, 9.9)	7.1 (3.7, 10.1)	10.65 (5.3, 12.6)	5.8 (3.8, 11.1)	6.5 (4.4, 12.7)
Males (*N* = 94)					
NLRP3 (ng/dL)	9.4 (5, 11.2)	10 (6.9, 10.8)	11.2 (10.4, 12.3)	5.4 (4,12)	5.8 (5, 9.9)
Females (*N* = 106)					
NLRP3 (ng/dL)	5.1 (2.5, 8.4)	3.9 (3.3, 7.9)	6.2 (2.5, 9.9)	6.4 (3.6, 10.5)	8.1 (4.4, 15.4)

Note: Data are presented as median (Q1, Q3).

**Table 4:** Bi-variate correlation between NLRP3 and other variables according to gender.

Parameters	Males (94)		Females (106)	
	**r**	***p***	**r**	***p***
Age	−0.11	0.29	**0.20**	**0.04**
BMI	−0.18	0.08	**0.32**	**<0.001**
Waist	−0.12	0.26	**0.24**	**0.02**
WHR	0.04	0.72	**0.33**	**<0.001**
Systolic	−0.08	0.44	**0.22**	**0.02**
Diastolic	−0.15	0.14	0.18	0.06
Cholesterol	−0.13	0.21	−0.13	0.19
FBG	−0.15	0.15	0.06	0.57
HDL-Cholesterol	−0.10	0.36	**−0.21**	**0.03**
Triglyceride	−0.01	0.97	0.12	0.23
Vitamin D	−0.04	0.74	0.09	0.36
Insulin	0.22	0.08	0.21	0.07
HOMA-IR	0.14	0.27	0.22	0.06
Quicki	−0.15	0.23	−0.22	0.06
IL-18	−0.07	0.54	−0.06	0.58
TNF-α	−0.14	0.33	0.04	0.73
IL-1β	−0.08	0.49	−0.05	0.64
CRP	−0.17	0.1	−0.17	0.09

Note: The data represent the Pearson correlation analysis of circulating NLRP3 levels with other measured parameters according to genders. The non-normal continuous variables were normalized by log-transformation before conducting the analysis. A *p* < 0.05 was considered statistically significant. Statistically significant *p*-values have been indicated by bold font. BMI, FBG, HDL, HOMA-IR represent body mass index, fasting blood glucose, high-density lipoprotein and Homeostatic Model Assessment of Insulin Resistance respectively.

**Table 5:** Logistic regression analysis showing association of components of MetS with circulating NLRP3 levels divided into tertiles.

Males (94)					
		Tertile 1 3.90 (3.0,4.6)	Tertile 2 9.30 (7.2,10.1)	Tertile 3 12.30 (11.4,14.2)	*p* ^t^
Central Obesity	Model a	1	0.52 (0.2, 1.4), 0.21	0.42 (0.1, 1.2), 0.09	0.21
	Model b	1	0.55 (0.2, 1.6), 0.27	0.54 (0.2, 1.6), 0.28	0.44
	Model c	1	0.71 (0.2, 2.5), 0.60	0.59 (0.2, 2.2), 0.42	0.72
Hypertension	Model a	1	0.25 (0.1, 0.8), 0.02	0.67 (0.2, 1.8), 0.44	0.04
	Model b	1	0.25 (0.1, 0.8), 0.02	0.79 (0.3, 2.6), 0.67	0.04
	Model c	1	0.28 (0.1, 0.9), 0.03	0.88 (0.3, 2.6), 0.81	0.06
Hyperglycemia	Model a	1	0.43 (0.2, 1.2), 0.10	0.59 (0.2, 1.6), 0.31	0.25
	Model b	1	0.45 (0.2, 1.3), 0.45	0.71 (0.3, 2.0), 0.71	0.32
	Model c	1	0.49 (0.2, 1.4), 0.19	0.76 (0.3, 2.2), 0.61	0.42
Low HDL-C	Model a	1	0.94 (0.4, 2.6), 0.91	1.29 (0.5, 3.5), 0.61	0.81
	Model b	1	1.00 (0.4, 2.7), 0.99	1.49 (0.5, 4.2), 0.45	0.68
	Model c	1	0.99 (0.4, 2.7), 0.99	1.46 (0.5, 4.1), 0.48	0.69
Hypertriglyceridemia	Model a	1	0.43 (0.2, 1.2), 0.10	0.87 (0.3, 2.4), 0.79	0.21
	Model b	1	0.45 (0.2, 1.3), 0.15	1.35 (0.4, 4.2), 0.60	0.12
	Model c	1	0.51 (0.2, 1.6), 0.26	1.58 (0.5, 5.2), 0.45	0.16
Full MetS	Model a	1	0.30 (0.1, 0.9), 0.03	0.68 (0.2, 2.0), 0.49	0.06
	Model b	1	0.34 (0.1, 1.1), 0.07	0.51 (0.2, 1.7), 0.26	0.19
	Model c	1	0.34 (0.1, 1.2), 0.08	0.63 (0.2, 2.2), 0.47	0.21
**Females (106)**					
		**Tertile 1** **3.10 (1.9,3.4)**	**Tertile 2** **6.35 (4.6,7.8)**	**Tertile 3** **13.80 (11.5,19.4)**	***p*^t^**
Central Obesity	Model a	1	1.29 (0.5, 3.2), 0.58	3.61 (1.3, 10.2), 0.01	**0.02**
	Model b	1	1.17 (0.5, 2.9), 0.74	2.98 (1.1, 8.7), 0.04	**0.04**
	Model c	1	1.12 (0.5, 2.8), 0.78	2.37 (1.1, 7.2), 0.04	**0.04**
Hypertension	Model a	1	1.51 (0.5, 4.2), 0.43	2.52 (0.9, 6.9), 0.07	0.19
	Model b	1	1.13 (0.4, 3.3), 0.82	1.70 (0.6, 5.0), 0.33	0.57
	Model c	1	1.06 (0.4, 3.2), 0.91	1.51 (0.5, 5.6), 0.46	0.71
Hyperglycemia	Model a	1	0.77 (0.3, 2.0), 0.59	1.44 (0.6, 3.7), 0.49	0.43
	Model b	1	0.46 (0.2, 1.3), 0.14	0.70 (0.2, 2.1), 0.53	0.32
	Model c	1	0.44 (0.1, 1.3), 0.13	0.64 (0.2, 2.0), 0.45	0.31
Low HDL-C	Model a	1	0.98 (0.4, 2.4), 0.96	3.33 (1.1, 9.8), 0.03	**0.04**
	Model b	1	1.03 (0.4, 2.6), 0.94	3.73 (1.2, 11.4), 0.02	**0.03**
	Model c	1	1.04 (0.4, 2.6), 0.93	3.96 (1.2, 12.6), 0.02	**0.03**
Hypertriglyceridemia	Model a	1	1.96 (0.7, 5.1), 0.17	3.35 (1.2, 9.1), 0.02	0.05
	Model b	1	1.42 (0.5, 4.1), 0.52	2.11 (0.7, 6.3), 0.18	0.39
	Model c	1	1.38 (0.5, 4.0), 0.55	2.04 (0.7, 6.2), 0.21	0.45
Full MetS	Model a	1	1.20 (0.5, 3.1), 0.71	4.05 (1.4, 11.5), 0.009	**0.01**
	Model b	1	0.80 (0.3, 2.2), 0.62	2.43 (0.8, 7.7), 0.13	0.09
	Model c	1	0.70 (0.2, 2.1), 0.53	2.06 (0.6, 6.9), 0.24	0.15

Note: Data of the logistic regression analysis were presented as odds ratio, 95% confidence interval, associated *p*-value. Odds ratio for higher NLRP3 tertiles (2 and 3) was calculated by taking tertile 1 as reference (represented by value 1). *p*^t^ represents the *p*-value for the trend. Models a, b and c are univariate, + adjusted with age and + adjusted with BMI, respectively. A *p* < 0.05 was taken as significant. Statistically significant *p*-values have been indicated by bold font.

## Data Availability

The data concerning this study is available from the corresponding author on reasonable request.

## Supplementary Materials

The following are available online at https://www.mdpi.com/article/10.3390/jcm10153288/s1, Table S1: Anthropometric and biochemical characteristics of the study subjects; Table S2: Anthropometric and biochemical characteristics of the study groups on gender basis.
