# Supplementary material for: Sex-Specific Signature in the Circulating NLRP3 Levels of Saudi Adults with Metabolic Syndrome
Source: J Clin Med. 2021 Jul 26;10(15):3288. doi: 10.3390/jcm10153288 (PMC8347773; doi:10.3390/jcm10153288)
Supplement: Supplementary file 1 [file jcm-10-03288-s001.zip › jcm-1212718-SI.pdf]

Sex-specific Signature in the Circulating NLRP3 Levels of Saudi Adults with Metabolic Syndrome

Nasser M. Al-Daghri, Kaiser Wani, Hind AlHarthi, Amani Alghamdi, Abdullah M. Alnaami, Sobhy M. Yakout

Table S1. Anthropometric and biochemical characteristics of the study subjects

Parameters	Non-MetS	MetS	P
N (F/M)	101 (50/51)	99 (56/43)	0.33
Age (years)	35.49 ± 8	41.94 ± 7.3	**<0.01**
BMI (kg/m²)	28.3 ± 6.6	31.87 ± 5.1	**<0.01**
Waist (cm)	87.43 ± 14.7	108.05 ± 14.5	**<0.01**
WHR	0.84 ± 0.1	0.99 ± 0.2	**<0.01**
Systolic (mmHg)	116.63 ± 12	130.6 ± 17.1	**<0.01**
Diastolic (mmHg)	71.01 ± 9.4	78.99 ± 11.2	**<0.01**
T.Chol (mmol/l)	5.22 ± 0.9	5.46 ± 1.6	0.19
FBG (mmol/l)	5.23 ± 0.9	7.19 ± 3.3	**<0.01**
HDL- C(mmol/l)	1.24 ± 0.3	1.02 ± 0.3	**<0.01**
Trigly. (mmol/l)	1.19 (0.9,1.5)	2.33 (1.9,2.8)	**<0.01**
Vitamin D [illegible]	42.39 (28.5,59.8)	41.95 (25.8,64.9)	0.77
Insulin (μU/ml)	8.06 (4.2,14.4)	17.28 (7.1,45.1)	**<0.01**
HOMA-IR	1.75 (1,3.8)	6.31 (2,11.6)	**<0.01**
Quickl	0.63 (0.5,0.7)	0.46 (0.4,0.6)	**<0.01**
IL-18(pg/ml)	64.09 (36.6,88.6)	48.59 (34,72.5)	0.17
TNF-α (pg/ml)	0.46 (0.2,1.2)	1.44 (0.8,1.8)	**<0.01**
IL-1β (pg/ml)	0.5 (0.4,0.9)	1.37 (1,2.9)	**<0.01**
CRP (μg/ml)	2.01 (0.59,4.2)	4.62 (1.7,6.2)	**<0.01**
NLRP3 (ng/dl)	8.65 (3.9,11.2)	6.35 (4.0,11.8)	0.82

Note: Data is presented as frequency, mean ± standard deviation and median (Q1, Q3) for categorical, normal continuous and non-normal continuous variables respectively. The statistical differences in each variable between the three groups, calculated by appropriate statistical tests, are presented as p-value.

Table S2. Anthropometric and biochemical characteristics of the study groups on gender basis

Parameters	Non-MetS (N=51)	MetS (N=43)	p	Non-MetS (N=50)	MetS (N=56)	p
		Males (94)			Females (106)	
Age (years)	35.27 ± 7.8	41.67 ± 7.2	**<0.01**	35.7 ± 8.2	42.14 ± 7.4	**<0.01**
BMI (kg/m^2)	26.61 ± 5.6	30.73 ± 4.3	**<0.01**	30.03 ± 7	32.75 ± 5.5	**0.03**
Waist (cm)	92.04 ± 16.9	110.34 ± 15	**<0.01**	82.72 ± 10.2	102.21 ± 15.1	**<0.01**
WHR	0.91 ± 0.1	1 ± 0.1	**<0.01**	0.76 ± 0.1	0.98 ± 0.2	**<0.01**
Sys (mmHg)	119.8 ± 12.3	130.49 ± 11.8	**<0.01**	113.4 ± 10.9	130.68 ± 20.4	**<0.01**
Dias (mmHg)	69.69 ± 9.4	76.58 ± 10.2	**0.001**	72.36 ± 9.4	80.84 ± 11.7	**<0.01**
T.Chol (mmol/l)	5.24 ± 0.9	5.42 ± 1.5	0.49	5.2 ± 1	5.5 ± 1.7	0.27
FBG (mmol/l)	5.22 ± 0.5	7.76 ± 3.7	**<0.01**	5.23 ± 1.2	6.75 ± 3	**0.001**
HDL-C(mmol/l)	1.13 ± 0.2	0.87 ± 0.2	**<0.01**	1.34 ± 0.3	1.12 ± 0.3	**<0.01**
Trigly.(mmol/l)	1.33 (0.9,1.7)	2.42 (2,3.4)	**<0.01**	1.07 (0.8,1.5)	2.21 (1.8,2.6)	**<0.01**
Vit.D (nmol/l)	44.97 (31.6,61.5)	36.01 (24.5,55.3)	0.05	33.28 (23,56.2)	50.58 (27.1,73.8)	**0.04**
Insulin (μU/ml)	11.2 (6.1,17.7)	18 (6.7,52)	0.93	5.72 (3.4,10.8)	17.28 (7.1,36.8)	**<0.01**
HOMA-IR	2.77 (1.6,4.2)	6.91 (1.8,14.9)	**0.01**	1.33 (0.7,2.4)	5.2 (2.3,9.6)	**<0.01**
Quickl	0.56 (0.5,0.6)	0.46 (0.4,0.6)	**0.01**	0.68 (0.6,0.8)	0.48 (0.4,0.6)	**<0.01**
IL-18(pg/ml)	40.07 (26,63.5)	54.05 (40.3,89.9)	**0.008**	77.59 (61.9,100.8)	42.18 (32.4,66.9)	**<0.01**
TNF-α (pg/ml)	0.21 (0.1,0.3)	1.31 (0.9,1.6)	**<0.01**	0.86 (0.4,1.5)	1.54 (0.7,1.9)	**0.007**
IL-1β (pg/ml)	0.43 (0.2,0.6)	1.34 (1.1,3.4)	**<0.01**	0.78 (0.4,1.6)	1.37 (1,2.7)	**<0.01**
CRP (μg/ml)	1.81 (0.74,4.09)	4.62 (1.9,6.2)	**0.001**	2.37 (0.5,4.5)	4.54 (1.3,6.2)	**0.009**
NLRP3 (ng/dl)	10.4 (8.3,11.4)	5.7 (4.0,11.3)	**0.014**	4.6 (3.3,9)	7.6 (3.7,13.7)	**0.04**

Note: Data is presented as frequency, mean ± standard deviation and median (Q1, Q3) for categorical, normal continuous and non-normal continuous variables respectively. The statistical differences in each variable between the three groups, calculated by appropriate statistical tests, are presented as p-value.
